# Use of Melatonin in Oxidative Stress Related Neonatal Diseases

**DOI:** 10.3390/antiox9060477

**Published:** 2020-06-02

**Authors:** Gabriella D’Angelo, Roberto Chimenz, Russel J. Reiter, Eloisa Gitto

**Affiliations:** 1 Neonatal and Pediatric Intensive Care Unit, Department of Human Pathology in Adult and Developmental Age “Gaetano Barresi”, University of Messina, 98125 Messina, Italy; egitto@unime.it; 2 Unit of Pediatric Nephrology and Rheumatology with Dialysis, Department of Human Pathology in Adult and Developmental Age “Gaetano Barresi”, University of Messina, 98125 Messina, Italy; rchimenz@unime.it; 3 Department of Cellular and Structural Biology, University of Texas Health Science Center at San Antonio, San Antonio, TX 40729, USA; REITER@uthscsa.edu

**Keywords:** oxidative stress, preterm newborn, infant, melatonin

## Abstract

Reactive oxygen species have a crucial role in the pathogenesis of perinatal diseases. Exposure to inflammation, infections, or high oxygen concentrations is frequent in preterm infants, who have high free iron levels that enhance toxic radical generation and diminish antioxidant defense. The peculiar susceptibility of newborns to oxidative stress supports the prophylactic use of melatonin in preventing or decreasing oxidative stress-mediated diseases. Melatonin, an effective direct free-radical scavenger, easily diffuses through biological membranes and exerts pleiotropic activity everywhere. Multiple investigations have assessed the effectiveness of melatonin to reduce the “oxygen radical diseases of newborn” including perinatal brain injury, sepsis, chronic lung disease (CLD), and necrotizing enterocolitis (NEC). Further studies are still awaited to test melatonin activity during perinatal period.

## 1. Oxidative Stress and Newborn

Oxidative stress (OS) was first defined by Sies as the imbalance between oxidants and antioxidants, favoring oxidants, which could lead to damage [1]. Free radicals (FRs), chiefly reactive oxygen species (ROS) and reactive nitrogen species (RSN), are usually produced during oxidative phosphorylation in the mitochondria. They are highly unstable, and formation is typically controlled by various compounds called antioxidant agents, both enzymatic (such as superoxide dismutase, glutathione peroxidase, and catalase) and non-enzymatic, mainly represented by vitamin E [1,2]. The anti-oxidative defense system is constituted by all these protective molecules. When hypoxic, hyperoxic, ischemic, and inflammatory events occur, the antioxidant system becomes insufficient and FRs, produced in a large amount, rapidly react with DNA, lipids, and proteins to produce cellular damage up to the death of the cell itself. ROS, mainly represented by superoxide anions (O^2−^), hydroperoxyl radicals, and hydroxyl (OH) radicals, are highly toxic for mitochondria, albeit the prime location for production; mitochondrial dysfunction and ROS production therefore augment each other [3].

Newborns are more vulnerable to OS, and, as a direct consequence of this, to OS damage. In passing from an intrauterine to extrauterine environment, the fetus moves from an intrauterine 20–25 mm Hg oxygen tension (PO_2_) to 100 mm Hg PO_2_ [4]. On the other hand, due to the low intrauterine PO_2_ levels, endogenous antioxidant systems in newborns are not properly calibrated to face this sudden change in O_2_ levels. As a result of this, a normal pregnancy leads to a significant increase of OS, whilst a complicated pregnancy produces an additional charge of ROS that requires a major antioxidant action that, often, the newborn is unable to provide. Moreover, both OS and nitrosative stress are highly dangerous for the newborn brain due to the immature cerebral white matter, mainly composed of promyelinating oligodendrocytes (O4 and O1), being highly vulnerable to FRs which, indeed, induce loss, leading to a decreased population of mature oligodendrocytes and to hypomyelination [5].

It is suspected that premature infants have an underdeveloped antioxidant defense system [6]. Therefore, newborns, especially if preterm, more commonly experience oxidative damage-mediated FR, because:

(a) At birth time, because of the passage from intrauterine to extrauterine life, newborns are exposed to the hyperoxic challenge. This event appears to be critical especially for newborns requiring additional oxygen in the delivery room, during resuscitation.

(b) Infections are more probably in newborns, above all if preterm.

(c) Elevated free iron levels augment the Fenton reaction, which leads to highly toxic radical synthesis [7].

Due to high susceptibility to OS, “free radicals disease” was coined to embrace a series of typical prematurity-related diseases where oxidative damage plays a defining role.

### 1.1. Oxidative Stress and Neonatal Lung

OS represents a frequent outcome for many events, including hyperoxia, inflammation, and mechanical ventilation, which play a part in sustained lung injury and cause impaired lung function [8]. OS leads to damage to the respiratory epithelium, surfactant inactivation, and an inflammatory status; consequently, a rise in the demand of mechanical ventilation is recorded. Moreover, the earlier preterm infants are exposed to OS, the more severe long-term respiratory outcomes can occur. Literature evidence reveals that mechanical ventilation, inducing repeated alveolar collapse and re-expansion and/or end-inspiratory alveolar over-stretching, impacts the balance across the alveolo-capillary membrane and hinders the integrity of the epithelium, endothelium, and surfactant system. The production of FRs initiates from diverse sources and may lead to oxidative lung injury in an at-risk neonate in the presence of immature antioxidant defenses. In the past few decades, data have indicated that OS is involved in the development of bronchopulmonary dysplasia (BPD) in preterm newborns; human studies have demonstrated a quantitative rise in oxidative damage to lipids and proteins in lung tissue and a reduction in levels of antioxidants in the biological fluids of ventilated preterm infants [7].

### 1.2. Oxidative Stress and Brain Injury

One of the main contributors to mortality and morbidity in premature and term infants is injury to the fetal brain [9]. Perinatal asphyxia is widely considered a risk factor for neonatal arterial ischemic stroke (NAIS), and both NAIS involving hypoxia–ischemia and neonatal hypoxic-ischemic encephalopathy (HIE) can contribute via different pathways. Perinatal stroke is caused by an oxygen-depriving event—a hemorrhage may disrupt normal blood circulation or a clot may form, leading to a reduction in the flow of oxygenated blood (ischemia). In the same way, HIE is a progressive brain injury caused by caused by oxygen deprivation and limited blood flow [10].

The pathogenesis of brain injury is complex and caused by multiple events; early events in the cascade of brain injury are classified as either inflammation or OS due to the production of FRs. ROS can initiate pro-inflammatory cytokine release and microglial activation, with a consequent release of other FRs and pro-inflammatory molecules. Initial OS takes place within 30 min of asphyxia, but ROS up-regulation may continue for as long as 28 days after reperfusion. The steps which cells are forced to undergo are impaired cerebral oxidative metabolism, swelling, and the accumulation of extra-cellular excitatory amino acids, followed by a short recovery before secondary energy failure sets in. Following reoxygenation, reperfusion injury leads to neuron cell death by causing irritant toxicity, mitochondrial dysfunction, pro-inflammatory response, the activation of nitric oxide synthase, the synthesis of ROS, and intracellular Ca^2+^ accumulation [9].

The brain is highly sensitive to oxidative injury due to its high oxygen consumption, high levels of intracellular free iron and polyunsaturated fatty acids in the neuronal membranes, and low contents of enzymatic antioxidants, above all in premature infants [11]. The association between OS biomarkers assessed in the first hours of life and brain damage highlights the possibility to identify newborns at greater risk of HIE early. Presently, the only established treatment in the subacute phase of asphyxia-induced brain injury is therapeutic hypothermia. A combination of new strategies and antioxidant supplementation at a very early stage, as future neuroprotection in asphyxiated infants, might alter the long-term outcome of these infants [11].

### 1.3. Oxidative Stress and Neonatal Gut

OS has also been indicated in the pathogenesis of necrotizing enterocolitis (NEC) [12], characterized by the inflammation and bacterial invasion of the bowel wall. It responds to a multifactorial etiology including an immature gut barrier, enteral/parenteral feeding, and an inadequate perfusion of the gut that results in an end result characterized by OS, inflammation, and the necrosis of the neonatal bowel. Though a direct cause–effect relationship between OS and NEC in humans has not yet been demonstrated, high levels of OS markers have been measured in the cord blood of NEC affected babies; in particular, Aydemir et al. showed that preterm infants with NEC had significantly higher total oxidant levels and OS index rates compared with NEC-free controls, and higher levels of these parameters were associated with higher severities of NEC [13].

## 2. Melatonin

Melatonin (*N*-acetyl 5-methoxytryptamine) is a neurohormone secreted from the pineal gland that has a wide-ranging regulatory role. It is synthesized from the neurotransmitter serotonin and has been recognized as an “ubiquitously distributed and functionally diverse molecule” [14]. Diverse physiological functions of melatonin have been shown, including circadian and endocrine rhythm regulation, anti-inflammation, and its potent analgesic, and anxiolytic and antioxidant activity [7,14,15,16,17,18,19]. The use of melatonin in the treatment of sleep disturbances, as an oncostatic and analgesic molecule, as premedication in surgical children, or as adjuvant to sepsis treatment, is widely recognized [19,20,21,22]. Moreover, melatonin exerts immunomodulatory effects in allergic diseases (such as atopic eczema) and chronic obstructive pulmonary diseases [23]. In addition, Gitto et al. [24] demonstrated that melatonin acts as an analgesic antioxidant in preterm newborns who undergo painful procedures, including mechanical ventilation and endotracheal intubation, especially when an inflammatory component is involved.

Melatonin is a broad-spectrum anti-apoptotic, antioxidant, and potent FR scavenger [14,25,26]. Aerobic organisms persistently produce FRs, which, when generated in excess, cause cell and tissue damage [27].

In the past few years, numerous publications have established that melatonin is a broad-spectrum antioxidant: This molecule and its metabolites upregulate antioxidant enzymes (including glutathione reductase and glutathione peroxidases) and down-regulate pro-oxidant enzymes (lipoxygenases and nitric oxide synthases) [28,29]. Melatonin, therefore, reduces mitochondrial hydroperoxide levels, prevents lipid peroxidation (LPO), and re-establishes glutathione balance [30].

A protective effect of melatonin in animal models of sepsis has also been highlighted [31,32], indicating a possible beneficial effect of this indolamine in septic patients, through the suppression of OS, nitrosative stress (NS), inflammation, and mitochondrial dysfunction.

When compared to normal age-matched patients, critically ill children show high endogenous melatonin levels, probably to counteract the increased risk of OS-mediated diseases [33]. Conversely, pregnant women affected by preeclampsia have shown reduced melatonin levels [34], suggesting that low levels of melatonin might account for the suppressed antioxidant capacity found in preeclampsia [35,36,37]. Melatonin synthesis has been found in the placenta, and villous trophoblasts have the classic transmembrane receptors for the indole, MT1 and MT2, suggesting a paracrine, autocrine, and/or intracrine role for melatonin in the human placenta and supporting the hypothesis that the increased maternal serum melatonin levels recorded during pregnancy originate from the placenta [38]. Melatonin detected in the fetus originates from the mother. It is influenced by circadian fluctuations, and levels rise slowly from 24 weeks of gestation, reaching higher levels in the third trimester. They return to baseline levels from the day two of puerperium. During the last trimester of pregnancy, the fetus develops a biological clock that is influenced by maternal circadian rhythms with variations in behavior, hormonal levels, and sleep.

Three-to-five months may pass after birth before a full-term newborn produces melatonin, experiencing a transient deficiency in melatonin levels [39]. Preterm infant research has given unclear findings: reduced urine aMT6s levels have been described at birth [40], but other studies have reported decreased 6-sulfatoxy-melatonin urine concentrations for 13 weeks after birth in preterm infants when compared to term infants [41]. In addition, higher plasma melatonin levels have been described in term infants in comparison to preterm neonates [42]. The differences seen among preterm infants could be correlated to environmental luminosity, the specificity and sensitivity of assays adopted in various biological samples, and/or drug interactions [43]. At 8–12 weeks after a term delivery, melatonin rhythmicity is established [7,44].

Melatonin is usually orally administered, but many other potential routes of administration have already been identified [45]. The oral dose control of exogenous melatonin is difficult due to high interindividual variability that results from a low bioavailability and an extensive first pass metabolism [46]. Conversely, it has been reported that the intranasal administration of melatonin reports an approximate bioavailability of 105% [47]. Concerning innovative routes of administration during brain injury, Aridas et al. demonstrated in animal models that melatonin offers promise as a transdermal therapy to protect the brain, thanks to its antioxidant, anti-inflammatory, and anti-apoptotic properties. Authors have reported that both systemic and transdermal neonatal melatonin administration are able to significantly reduce neuropathology and encephalopathy characteristics associated with perinatal asphyxia. Therefore, a novel use of melatonin, through a melatonin patch applied soon after birth could lead to better outcomes in newborns affected by asphyxia, above all for births in countries where resources are scarce [48].

## 3. Use of Melatonin in Neonatal Period

Melatonin administration during the late fetal and early neonatal period might provide health benefits, improve the quality of life, and limit complications occurring prior to and shortly after delivery. Accordingly, much evidence supports the use of melatonin in perinatal conditions, including asphyxia, chronic lung disease (CLD), respiratory distress syndrome (RDS), surgical processes, and sepsis [22,49,50,51,52]. Moreover, as preterm infants show a deficiency in melatonin levels, the administration of the drug might guarantee the needed levels to assure health [53,54].

### 3.1. Melatonin and Lung Damage

Several etiologic factors have been identified as causes of lung injury in the neonatal period such as genetic, hemodynamic, metabolic, nutritional, mechanical, and infectious mechanisms (inflammatory placental disorders and chorioamnionitis). Acting in a synergic manner, all these events lead to an increased synthesis of FR which, in turn, induces OS-mediated tissue damage.

Particularly, studies investigating airway inflammation [55] have revealed that oxygen therapy, despite being crucial in treating respiratory disorders, can both induce an inappropriate production of ROS/RNS in the airways and cause damage to endothelial and epithelial cell barriers, therefore contributing to the development of CLD [56].

In this regard, melatonin appears to be an effective antioxidant that limits radical-induced damage. In an in vivo study, authors showed that hyperoxia-induced increases of nitrite/nitrate, myeloperoxidase, and malondialdehyde (MDA) levels were prevented by melatonin and weakened the reduction of antioxidant enzymes [57]. Pan et al. demonstrated that the nocturnal administration of 4 mg/kg melatonin prevented the reduction of interstitial fibrosis and the total number of alveoli caused by CLD, as evaluated by collagen fiber staining and semi quantitative morphological indices of radical alveolar counts [57]. On the other hand, FRs can be also produced by high pressure generated during mechanical ventilation; this event accounts for CLD development in premature infants who, although not exposed to supplemental oxygen, experience lung damage due to the ventilator strategies used [56,58]. Accordingly, mechanically ventilated newborns with RDS who received melatonin showed a significant reduction in the serum levels of inflammatory cytokines (tumor necrosis factor (TNF)-α, interleukin (IL)-6, and IL-8) in comparison to infants mechanically ventilated but not receiving melatonin. Moreover, higher serum inflammatory cytokines levels were noted in infants who developed CLD [59]. Therefore, authors, assessing the antioxidant and anti-inflammatory effects of melatonin, proposed it as a potential molecule in preventing CLD onset in mechanically ventilated newborns [59].

Since melatonin counteracts ROS and RNS release [60], it might also prevent ROS- and RNS-induced tissue damage occurring in the early stages of inflammation. Melatonin, by inhibiting neutrophil and leukocyte recruitment into the lung, might provide a further protection for pulmonary tissue from FR, such as myeloperoxidase, produced by inflammatory cells [61,62]. Specifically, ligand-receptor binding reaction could be one of the mechanisms by which melatonin counteracts lung damage, as is suggested by the upregulation of melatonin receptor mRNA in lipopolysaccharide-mediated lung injury [62].

### 3.2. Melatonin and Brain Injury

The fetal and neonatal brain are highly susceptible to OS due to their poorly developed innate defense systems, above all due to low levels of glutathione peroxidase, catalase, and vitamin E [63]. Innate immunity, which is mediated by specific cellular and molecular programs and signaling, contributes to pathology in brain damage in response to OS. Numerous mediators of innate immunity play a dual role in pathology and physiology in the developing brain. Toll-like receptors (TLR)s 1–9 mRNA expression has been identified in developing murine brains. TLR3 activation hinders neural progenitor cell proliferation during brain maturation, and the inhibition of TLR-3 in fetal life modifies brain development through IL-6 signaling. TLR-1, -2, and -7 are upregulated 24 h after any injury, and TLR-2, -3, -6, -7, and -9 are upregulated 72 h after hypoxic-ischemic injury; meanwhile, TLR-5 is downregulated in the first 24 h [64].

It has been documented that both OS and neuro-inflammation are early and crucial events in the cascade of brain damage. Consequently, to prevent the long-term sequelae of this oxidative injury, an early diagnosis with specific biomarkers of OS is necessary, allowing for the delineation of new potential therapeutic treatments. Currently, a promising neuroprotective therapeutic strategy consists of blocking these mechanisms of brain injury. The additive effect of another molecule, docosahexaenoic acid, on hypothermia has also been investigated to reduce the degree of OS in the white matter, the prefrontal cortex, and the hippocampus in the brains of hypoxic-ischemic piglets. Authors reported that therapeutic hypothermia diminished lipid peroxidation in the white matter but not in the cortical gray matter, and it weakened the reducing effect of docosahexaenoic acid on lipid peroxidation in the cortex [65].

Currently, melatonin is thought of as a neuro-protective drug with great potential for acute and chronic brain injuries, although the specific function of a protective agent against newborn hypoxic-ischemic brain injury has not yet been completely understood [66]. This molecule is a potential non-toxic, natural neuroprotective agent in decreasing brain damage and long-term sequels, either as an add-on or stand-alone therapy. It also has an excellent safety profile in high concentrations; it can easily cross the blood–brain barrier and possesses anti-inflammatory and antioxidant actions [67].

In the pathogenesis of hypoxia–ischemia, the time of injury and the timing of treatment play crucial roles. The deprivation of glucose and oxygen causes an initial energy failure and induces multiple biochemical events that, by causing cell dysfunction-to-death, results in neurological damage featuring the HIE. Mitochondrial dysfunction carries out a pivotal role in the delayed mechanisms of brain cell injury, including the activation of apoptosis and secondary energy failure [68]. An initial drop in high-energy phosphates may result in an acute influx of Na^+^, water, and Cl^−^, with consequent necrosis in the severe injury; in a less severe insult, it causes membrane depolarization followed by a cascade of excitotoxicity and OS, resulting a delayed cell death, mainly apoptosis [68].

While current standard treatment includes therapeutic hypothermia for neonates with moderate-to-severe HIE, this approach is only effective in part, and it has been demonstrated that >40% of cooled newborns die or survive with impairment. Therefore, there is much interest in identifying additional promising therapeutic drugs to enhance the beneficial effects of therapeutic hypothermia [69,70].

Developing brain tissue, both in fetal and neonatal age, is highly susceptible to oxidative insult, and the strong FR-scavenging properties of melatonin provide a fundamental neuroprotective mechanism, ameliorating secondary cerebral energy failure and apoptotic damage [71,72]. Sinha et al. demonstrated that melatonin mediates its neuroprotective effect in murine models of newborn with brain injury through the suppression of mitochondrial cell death pathways, the restoration of MT1 receptors, and microglial activation [73].

The neuroprotective effects of melatonin have been reported by several authors in in vivo studies [74,75,76,77,78,79]. Melatonin administration in preterm and near-term fetal sheep, which underwent intrauterine asphyxia via umbilical cord occlusion, reduced OS [75] and attenuated cell death, as well as a reduced inflammatory response, in the fetal brain [76]. Following melatonin administration, lower fraction levels and caspase-3 activation were observed in an in vivo model [77]. Signorini et al. proved increased concentrations of desferrioxamine-chelatable free iron in the cerebral cortex related to hypoxia with the consequent development of OS, which may be prevented by the administration of melatonin [78]. Additionally, melatonin administration in rats with acute neonatal hemorrhagic brain injury gives protection against the post-hemorrhagic consequences of brain atrophy. Interestingly, in juvenile rats, melatonin was also found to improve clinical outcomes, such as cognitive and sensorimotor dysfunction [79].

Firstly, melatonin administered to human-asphyxiated newborns induced the reduction of serum oxidative molecules levels, such as MDA and nitrite/nitrate, within the first 6 h of life [49]. In particular, authors orally administrated a total of 80 mg of melatonin (eight doses of 10 mg, each separated by 2-h intervals) to ten asphyxiated newborns. One blood sample was collected before melatonin was administered after a blood sample was collected, and two further blood samples, at 12 and 24 h, were collected after administering indolamine. It was found that serum MDA and nitrite/nitrate concentrations in newborns with asphyxia before treatment were significantly higher than those in infants without asphyxia. In the asphyxiated newborns who had been administered melatonin, there was a signification drop in MDA and nitrite/nitrate levels at both 12 and 24 h. Authors confirmed the antioxidant properties of the melatonin, along with its ability to improve mitochondrial electron transport [49].

Though the only proven treatment for HIE in newborns is hypothermia, the addition of melatonin supplementation has been hypothesized to provide a major neuroprotective effect and, consequently, improve neonatal outcome. Accordingly, by using clinically relevant magnetic resonance spectroscopy biomarkers in an in vivo model, Robertson and colleagues confirmed the neuroprotective effects of melatonin in association with therapeutic hypothermia, as assessed by an improvement in cerebral energy metabolism and reduction in brain injury [80]. Subsequently, Aly et al. in a pilot clinical trial, studied the effect of melatonin on the biochemical, clinical, radiological, and neurophysiological outcomes of neonates with HIE and examined the feasibility and efficacy of enterally administered melatonin (five daily doses of 10 mg/kg per day consisting of melatonin tablets crushed and dissolved in distilled water) to those neonates who were receiving whole-body therapeutic hypothermia [81]. Using electroencephalography and magnetic resonance imaging (MRI) analyses, authors showed that the combination of the early administration of melatonin along with therapeutic hypothermia in infants with moderate-to-severe HIE was effective in reducing OS and improving survival without neurological or developmental abnormalities in the melatonin group, with improved results regarding number of seizures on follow-up electroencephalography and white matter abnormalities in MRI [81].

The efficacy, safety, and pharmacokinetics of 5 and 15 mg/kg/24 h melatonin administered at 2 and 26 h after hypoxia–ischemia with cooling in an in vivo model was recently demonstrated by Robertson et al. The same authors also concluded that putative plasma therapeutic melatonin levels were ~15–30 mg/L. Therefore, obtaining therapeutic plasma melatonin levels earlier may optimize protection by targeting the initial events of reperfusion insult [82]. Subsequently, Robertson and colleagues assessed that 18 mg/kg melatonin rapidly administered over 2 and 1 h after hypoxia–ischemia with cooling from 1–13 h was safe, and therapeutic levels were achieved within 3 h; in addition, in parallel, an increased hypothermic neuroprotection was guaranteed. The efficacy of the early administration of melatonin, combined with cooling, was also assessed by a faster aEEG recovery 19 h after hypoxia–ischemia, improved brain energy metabolism and reduced quantitative cell death in eight brain regions (TUNEL-positive cells), especially in the most severely damaged regions. All these findings confirmed that melatonin was a new, safe neuroprotective drug agent that increases the beneficial effects of therapeutic hypothermia where target therapeutic levels are achieved at ~2 h following hypoxic-ischemic event [83].

There is a strong evidence to support integration with melatonin as a neuroprotective treatment for premature and term hypoxic-ischemic brain injury, whether administered as a prophylactic or post-insult treatment, through antioxidant and anti-inflammatory actions. Therefore, this indolamine has many attributes that make it a promising therapy in newborns [84]. Furthers clinical research is needed to evaluate its real benefits in a neonatal population and to support its use in routine practice in asphyxiated infants.

### 3.3. Melatonin and Sepsis

Sepsis is one of the major causes of neonatal morbidity and mortality. Along with inflammation, OS is involved in detrimental pathways activated during neonatal sepsis, which inevitably leads to organ dysfunction and death. IL-6 and IL-8 start a redox cascade during sepsis in newborns that is characterized by multiple noxious processes such as the activation of gene expression that leads to the amplification of inflammation and OS, direct cell damage induced by ROS, and mitochondrial dysfunction. It has been suggested that, after the release of pro-inflammatory cytokines, diverse OS-related pathways are activated through different mechanisms, triggering the start of a self-maintaining “sepsis redox cycle” that ends in mitochondria impairment and cell oxidative damage [85,86].

The elevated production of FRs and pro-inflammatory cytokines, together with the innately low levels of plasma antioxidants in newborns, has been indicated in the complications and pathogenesis of neonatal sepsis. The immature immune system of newborns increases susceptibility to multiple recurrent infections. Moreover, newborns are more susceptible to OS that occurs during sepsis than adults because of reduced levels of endogenous antioxidants, e.g., beta-carotene, vitamin E, and sulfhydryl groups. In neonatal sepsis, both OS-related pathways and antioxidant defenses would seem to be induced [86]. In line with these findings, in 70 septic newborns with a mean gestational age of 36 weeks, the total oxidant state (TOS) and the total antioxidant state (TAS) were both elevated during the pretreatment period compared to healthy newborns, and the OS index (OSI) and the percentage ratio of TOS/TAS, were also elevated [87], ultimately assessing the prevalence of OS pathways on antioxidant defense.

FR-induced OS has been correlated with the severity of sepsis, as well as sepsis-related mortality and morbidity. Thus, an analysis was undertaken in our study to assess the effect of a new therapy with melatonin in order to add some important insights on a promising clinical benefit of melatonin in neonatal sepsis.

In septic neonates, the serum levels of lipid peroxidation products MDA and 4-hydroxylalkanals (4-HDA) were tested before and after 1 and 4 h of melatonin administration. Sepsis-related serum parameters and clinical status were assessed at 24 and 48 h after melatonin use. Gitto et al. founded that serum MDA and 4-HDA levels were significantly elevated in septic neonates compared to healthy infants; conversely, newborns receiving melatonin showed a significant decrease in MDA and 4-HDA levels, similarly to a control group. After 24–48 h, clinical outcome and sepsis-related serum parameters had also improved after melatonin administration. In summary, the authors demonstrated the effectiveness of melatonin in septic newborns by diminishing the levels of lipid peroxidation products and improving the clinical status of patients [22].

### 3.4. Melatonin and NEC

The development of the immaturity of intestinal barrier function, prematurity, immune system, feeding with formula milk, hypoxic-ischemic injury, and colonization by pathologic bacteria and inflammatory mediators are widely recognized risk factors for NEC, a gastrointestinal surgical disease in premature neonates [88]. Firstly, an initial stress induces the release of pro-inflammatory agents, e.g., platelet activating factor and, subsequently, inflammatory cytokines, e.g., TNF-α and IL-6. Successively, the recruitment of activated polymorphonuclear leukocytes and the abnormal generation of ROS occur. It would appear that ROS also contribute to tissue damage via the peroxidation of unsaturated lipids which, in turn, leads to intestinal hypoxia/reoxygenation injury. A synergistic effect of these cytokines triggers a cascade of events that leads to an eventual breakdown of the intestinal mucosal barrier and severe NEC in some cases [89]. Melatonin, apart from standard therapies, could be considered as a potentially safe approach to prevent and treat NEC in preterm infants. In an animal model, Guven et al. demonstrated that oxidative and nitrosative stresses are critical components of NEC-like injuries, and they further showed that melatonin significantly diminished the severity of NEC. In newborn rats, an NEC-like injury was induced by enteral formula feeding and exposure to hypoxia after cold stress at 4 °C and oxygen. It was found that 10 mg/kg melatonin given daily for three days after the first day of the NEC procedure led to a significant reduction in the severity of NEC, decreasing inflammatory cytokines and increasing antioxidant enzyme activities [90]. Therefore, it may be that antioxidant melatonin is useful in the management of newborns with NEC.

## 4. Conclusions

The role of FRs in the pathogenesis of several diseases of newborns, especially if preterm, is widely recognized. The susceptibility of newborns to OS-mediated damage supports the prophylactic use of antioxidants, such as melatonin, in preventing or reducing OS-mediated diseases. Multiple investigations have assessed the effectiveness of melatonin to reduce the “oxygen radical diseases of newborn” including perinatal brain injury, CLD, NEC, and sepsis. Further studies are still awaited to test melatonin activity during the perinatal period.
